# Experiences of Women Who Opt for a Planned Home Birth After a Previous Hospital Birth: A Qualitative Study

**DOI:** 10.3390/nursrep16040147

**Published:** 2026-04-21

**Authors:** Trinidad Maria Galera-Barbero, Vanesa Gutierrez-Puertas, Helder Jaime Fernandes, Blanca Ortiz-Rodriguez, Alba Sola-Martinez, Lorena Gutierrez-Puertas

**Affiliations:** 1 Department of Midwifery, University Hospital Torrecardenas, 04009 Almeria, Andalusia, Spain; trinidadm.galera.sspa@juntadeandalucia.es; 2 Department of Hematology, Torrecardenas University Hospital, 04009 Almeria, Andalusia, Spain; blanca.ortiz.sspa@juntadeandalucia.es; 3 Department of Nursing, Physiotherapy and Medicine, Faculty of Health Science, Universidad de Almería, 04120 Almeria, Andalusia, Spain; lgp524@ual.es; 4 Research Group PAIDI-TIC 019 “Electronic Communications and Telemedicine”, Universidad de Almeria, 04120 Almeria, Andalusia, Spain; 5 Research Centre for Active Living and Wellbeing (LiveWell), Polytechnic Institute of Bragança, 5300-253 Bragança, Portugal; helder@ipb.pt; 6 Department of Midwifery, Hospital La Inmaculada, 04600 Almeria, Andalusia, Spain; alba.sola.sspa@juntadeanadalucia.es; 7 Research Group PAIDI-HUM 061 “Experimental and Applied Neuropsychology”, Universidad de Almeria, 04120 Almeria, Andalusia, Spain

**Keywords:** planned home birth, hospital birth, women, midwives, private practice, nursing, qualitative research

## Abstract

**Background/Objective**: In Spain, 99% of births occur in hospital settings, and planned home birth is neither funded nor regulated by the Public Health System. Despite growing interest in this birth option, qualitative evidence exploring the experiences of women who opt for a planned home birth after a previous hospital birth remains scarce, particularly in contexts where this practice is not integrated into the healthcare system. This study aimed to explore the perceptions and experiences of Spanish women who opted for a planned home birth following a previous hospital birth, focusing on the reasons that motivated this decision and the care received during the process. **Methods**: A qualitative descriptive design was employed. Semi-structured interviews were conducted between July and December 2025 with 19 women who had experienced a planned home birth in Spain after a previous hospital birth. Data were analysed using inductive thematic analysis following Braun and Clarke’s approach. The study adhered to the Standards for Reporting Qualitative Research (SRQR). **Results**: Three main themes emerged: (1) motives related to choosing a planned home birth, including negative hospital experiences characterised by loss of autonomy, medicalisation of birth without consent, and fragmented care; (2) seeking a physiological and humanised birth, reflecting women’s desire for empowerment, control, and a transformative experience, alongside barriers such as lack of professional support and financial burden; and (3) the need to increase visibility and establish regulation, highlighting demands for professional training, dissemination strategies, and integration of planned home birth into the Public Health System to ensure equitable access. **Conclusions**: Women who opted for a planned home birth after a hospital experience reported highly positive and empowering outcomes. However, the absence of regulation, professional support, and public funding creates significant inequalities. Integrating planned home birth into the Public Health System, educating healthcare professionals, and developing strategies to increase the visibility of planned home births are essential to guarantee women’s right to choose where they give birth.

## 1. Introduction

Planned home birth has become a topic of growing interest, as the number of women who opt for a planned home birth has increased in recent years [1]. The term planned home birth refers to women who decide to give birth at home accompanied by a registered midwife [2]. The midwife informs the woman about the available birth setting options, conducts the assessment to determine eligibility for planned home birth, carries out home visits, attends the normal birth at home, and provides postpartum care to both the mother and the newborn [3].

In several countries, the maternal healthcare system provides the option of planned home birth or hospital birth for low-risk women, thereby promoting shared decision-making [4]. However, the Spanish Public Health System (PHS) only offers women the option of giving birth in an obstetric unit [5]. In countries where planned home birth is funded by the PHS, the rates of planned home births range from 30% in the Netherlands [6] to 0.7% in Australia [7]. In Spain, the vast majority of births take place in the hospital setting, with only 0.3% of births occurring at home [8], making hospital birth the cultural norm [9].

Planned home birth for low-risk women, defined as those with a singleton, full-term, cephalic presentation pregnancy, without obstetric complications or significant maternal comorbidities, and whose labour began and progressed without any warning signs [2], has been associated with several potential advantages compared to hospital birth [10]. Recent observational meta-analyses suggest that women who opt for a planned home birth are twice as likely to have a spontaneous delivery, and present a lower risk of caesarean section or instrumental delivery and a reduced risk of postpartum haemorrhage and foetal dystocia, as well as a decrease in medical interventions compared to hospital births [10,11]. Regarding neonatal morbidity and mortality, including Apgar score, neonatal asphyxia, and perinatal death, some studies report comparable outcomes between planned home birth and hospital birth in low-risk women when attended by registered midwives [12]. However, other studies have reported a slightly increased risk of neonatal mortality associated with planned home birth, particularly among nulliparous women [2,13]. These contradictory findings highlight the need for cautious interpretation of the available evidence [10,11].

In the provision of high-quality maternal care, the importance of involving women in decisions regarding their care, as well as considering their values and preferences regarding birth setting, has been widely emphasised [14]. Women have the right to choose where they give birth [15]. The birth setting has a significant impact on women’s experiences [16]. Some studies suggest that decisions about birth settings are influenced by maternity healthcare professionals and that women are not an integral part of the decision-making process [17,18]. Likewise, women face difficulties in obtaining the necessary support from healthcare professionals involved in the birth process when they opt for a planned home birth [19,20].

A recent systematic review suggests that women opt for a planned home birth to feel more comfortable in their own environment or to avoid unnecessary medicalisation of birth, seeking a physiological birth [21]. A physiological birth is defined as one that occurs driven by the innate capacity of the woman and the foetus, without unnecessary interventions that alter normal physiological processes [22]. However, this term lacks a universally accepted definition in the literature, which can lead to variations in its interpretation among healthcare professionals and women [16]. Women who opt for planned home births report better experiences associated with continuity of care [23] and participation in the decision-making process [24], regardless of whether they are transferred to the hospital during labour [25]. Likewise, newborns are more likely to be breastfed after birth [10] and to continue breastfeeding during the first six months of life [26]. Women who opt for a planned home birth report feeling safe and comfortable, given that they have access to a familiar environment and the support of a registered midwife [2,6], and overall regard the experience as satisfactory [12].

In Spain, women who decide on a planned home birth may face greater challenges associated with their decision to give birth at home compared to countries where planned home birth is integrated into maternity care programmes [26] or is funded by the PHS or covered by private insurance [5]. Women in Spain who opt for a planned home birth must privately contract the services of a midwife or take out private health insurance that covers some planned home birth services, such as prenatal education or postpartum care, but does not cover intrapartum care [9]. Midwives who perform planned home births practice privately as self-employed healthcare professionals; however, they may refer to the PHS when necessary, given that women retain the right to utilise available PHS resources, including prenatal testing, postpartum care, and access to emergency services [27].

Previous studies have explored women’s preferences regarding birth settings and their experiences in the decision-making process when opting for a planned home birth in countries where planned home birth is funded by the PHS [1,28]. The available evidence indicates that multiparous women, compared to primiparous women, are those who most frequently prefer a planned home birth [11,12]. However, women’s experiences in the decision-making process regarding planned home birth and the reasons that motivate them to change their birth setting have been scarcely explored. With regard to intrapartum care, studies suggest a positive impact on satisfaction and health outcomes in planned home births [29,30]. Similarly, no studies have been conducted in countries where planned home birth is not funded by the PHS that explore the experiences of women who opt for a planned home birth following a previous hospital birth. Therefore, this study sought to explore whether this is reflected in the decision to opt for a planned home birth. Qualitative research into the experiences of women who opt for a planned home birth following a previous hospital birth is virtually non-existent in countries where this option is neither integrated into nor funded by the Public Health System, which represents a significant gap in current knowledge. Based on the aforementioned evidence, the objective of this study was to explore the perceptions and experiences of Spanish women who opt for a planned home birth following a previous hospital birth, with regard to the reasons that motivated this decision and the care received during the planned home birth. This study provides new insights into the motivations and experiences of these women, helping to shed light on a little-explored reality and to guide PHS policies towards care that is more focused on women’s needs and preferences.

## 2. Materials and Methods

### 2.1. Design

The present study used a descriptive qualitative design using semi-structured interviews for data collection to address the following research question: what are the experiences and perceptions of women who opt for a planned home birth after a previous hospital birth? This qualitative approach contributes to generating in-depth data on a poorly understood phenomenon by exploring the deep perceptions and experiences of women who opt for a planned home birth after a previous hospital birth experience, as well as understanding the reasons for choosing a planned home birth and their experience of the care received from midwives during the planned home birth [31]. Descriptive qualitative methods with thematic analysis were used as they are appropriate for providing descriptions of phenomena with sparse literature [32]. The Standards for Reporting Qualitative Research (SRQR) was used to report this study (Supplementary Materials) [33]. The study was conducted from July 2025 to December 2025.

### 2.2. Participants and Setting

The study was conducted with Spanish women who opted for a planned home birth after a previous hospital birth. In this study, a planned home birth was defined as a birth that was intentionally planned to take place at the woman’s home, attended by one or two registered midwives with specific training and experience in home birth care, in low-risk women (defined as a singleton, full-term pregnancy with cephalic presentation, without obstetric complications or significant maternal comorbidities), where a prior assessment of the home environment and proximity to a referral hospital (less than 30 min) had been carried out [11]. The classification of the place of birth was based on the initial intention at the onset of labour, regardless of where the birth ultimately took place. Of the 19 women, one was transferred to hospital due to a lack of progress in dilation. This classification criterion based on initial intention is consistent with the methodological recommendations found in the international literature on studies of planned home births. In Spain, the PHS only funds births in the hospital setting. Women who opt for a planned home birth must pay for a private service or take out private health insurance that covers some planned home birth services, such as prenatal education or postpartum care [5]. In order to provide this care, midwives must work privately, either in a group practice or independently [27]. Purposive sampling was used for the selection of the participants, to ensure geographic diversity in Spain to obtain enriching data related to the objective of the study. The recruitment followed a three-stage pathway: firstly, professional midwifery associations were contacted to identify midwives who attended planned home births; secondly, emails were sent to registered midwives who attended planned home births, identified through these associations, supplemented by searches on professional social media platforms; and finally, these midwives acted as intermediaries, facilitating access to eligible participants by informing women they had attended to during a planned home birth about the study and providing details so that those interested in participating voluntarily could contact the principal investigator. A total of 35 midwives who attended planned home births in 10 Spanish autonomous communities were contacted; of these, 18 agreed to collaborate as intermediaries in the recruitment process, identifying 25 women who met the inclusion criteria. Of these, 19 agreed to take part in the study and 6 declined, citing mainly a lack of availability (n = 3) or a lack of interest in participating (n = 3). The established inclusion criteria were as follows: having had a planned home birth in the last year in Spain; having had a previous hospital birth in the same country; being a native Spanish speaker; and providing informed consent to participate in the study. The sample size was determined based on the sufficiency of the information obtained, which allowed us to adequately address the research question and generate a rich and detailed understanding of women who opt for a planned home birth after a previous hospital birth. A total of 19 women participated in the study.

### 2.3. Data Collection

Data collection was conducted between July and December 2025 through semi-structured interviews. Researchers developed an interview protocol based on existing literature (Table S1) [1,12]. Nineteen interviews were conducted by researchers trained in qualitative research methods, with a moderator present to oversee and monitor each session. The interviews were carried out by the principal investigator, a midwife with specific training in qualitative research methods and previous experience in conducting semi-structured interviews in the field of maternal health. The interviews were conducted both in person (n = 13) and online via video conference (n = 6), depending on the participants’ geographical location and preference. The online interviews were conducted using the Google Meet platform, ensuring the same level of privacy, confidentiality and recording quality as the in-person interviews. Before starting the interviews, it was ensured that all participants met the inclusion criteria, an email was sent with a brief introduction to the study, its aim, and the contact details of principal investigator, and participants provided and signed their informed consent to participate. Interviews were digitally recorded with prior consent from the participants, who were assured that they would remain unidentified throughout the study. The interviews lasted between 45 and 60 min. The interviews were conducted between 2 and 6 months after the planned home birth (mean: 3.8 months). All interviews were conducted in Spanish, the native language of both the participants and the researchers, in order to ensure the natural expression of women’s experiences and perceptions. The data collection process employed an informed approach based on the concept of information power, prioritising the depth and richness of insights over the notion of reaching a predetermined point of saturation [34]. All transcripts were anonymised, and participants were given the opportunity to examine them prior to analysis to verify that all their contributions were accounted for. The coding and thematic analysis process was carried out entirely in Spanish, preserving the linguistic and cultural nuances of the original data. Once the analysis was completed and the definitive themes were established, the representative quotes selected for inclusion in the manuscript were translated into English by a bilingual researcher and verified through back-translation into Spanish by a second bilingual researcher to ensure the accuracy and fidelity of the translated quotes to the original meaning.

### 2.4. Data Analysis

**Figure 1:**
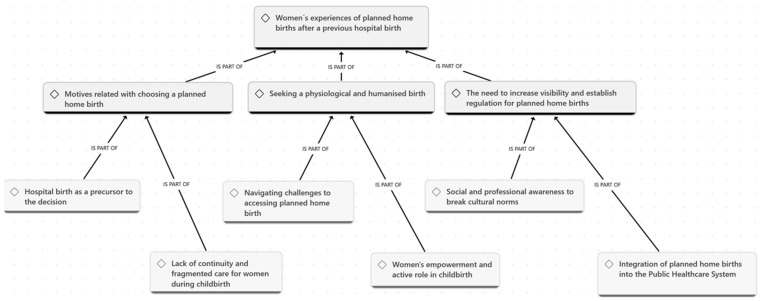
Concept map based on women’s experiences of planned home births after a previous hospital birth.

The audio-recorded interviews were transcribed verbatim using Microsoft Office Word 365 by two researchers. Subsequently, alphanumeric codes were assigned to each participant (P-X) to ensure privacy, confidentiality and anonymity. The data were analysed using ATLAS.ti Version 25.0.1 software (ATLAS.ti Scientific Software Development GmbH, Berlin, Germany), following the phases outlined by Braun and Clarke for inductive thematic analysis [34]: (1) the data analysis and familiarisation phase began during transcription and initial understanding, as well as reading the texts while noting initial ideas; (2) for systematic data coding, the text was re-read, and initial codes were generated from the dataset, which were grouped by meaning and patterns; (3) for theme generation, initial themes were identified, and a thematic map was created to find relationships between themes; (4) theme development and review was carried out to ensure consistency among codes grouped by themes; (5) the analysis, definitions and themes names were refined, and preliminary coding and themes were discussed and refined by the corresponding author and last author; (6) a report was prepared analysing the previously selected excerpts and relating this analysis to the research question for finalisation. The coding process was carried out iteratively; two researchers coded the data independently, and any discrepancies identified were resolved through discussion until a consensus was reached or, failing that, by consulting a third researcher. An audit trail was maintained throughout the analytical process, documenting decisions related to coding, the grouping of codes into subthemes and themes, and modifications made during discussion sessions between researchers, thereby ensuring the traceability and transparency of the analytical process. A conceptual map was developed encompassing the three main themes that illustrate the perceptions and experience of woman who opt for planned home birth after a previous hospital birth (Figure 1).

### 2.5. Ethical Considerations

The study proposal was approved by the Ethics Committee at the University of Almeria, Spain (EFM 444.25, July 2025), which followed the principles of the Declaration of Helsinki and subsequent modifications [35]. Before commencing the study, all participants were informed about the aim of the study and their right to withdraw their informed consent and decline to answer any questions that were asked, at any time, without retribution. Participants who decided to participate provided their written informed consent. They were afforded the continual option to withdraw at any moment while also ensuring preservation of their confidentiality and anonymity. In compliance with the General Data Protection Regulation (EU) 2016/679, all participants’ personal data were anonymised by assigning alphanumeric codes (P-1 to P-19) prior to storage and analysis. Audio recordings and transcripts were stored in a Google Drive folder protected by encryption and two-step authentication, with access restricted exclusively to the two researchers responsible for data analysis. Files will be retained for a period of five years following publication of the study and subsequently permanently deleted. No participant data (transcripts, quotes, personal information or any identifiable data) were accessed or processed by artificial intelligence tools.

### 2.6. Rigour and Reflexivity

The methodology and results of the study were assessed based on the recommendations for qualitative research reporting SRQR [33]. The trustworthiness of this study was ensured by adhering to the Lincoln and Guba criteria [36]. Transferability was addressed by providing a comprehensive account of the methods and data collection, as well as direct quotations in the presentation of the findings, Conformability was attained through the independent analysis of transcripts by the corresponding author and last author, followed by a collaborative session to compare, correlate and discuss emerging themes according to the guidelines established by Braun and Clarke [34] to ensure the validity and accuracy of the data. The discrepancies related to the study design, data analysis and conclusions were discussed among the research team members until a consensus was reached [37]. To minimise interviewer bias, the interviews were conducted by the principal investigator. With regard to reflexivity, the principal investigator is a midwife with clinical experience exclusively in a hospital setting, with no professional involvement in home birth care; this facilitated a more neutral stance during data collection and interpretation. However, prior to commencing the study, the research team reflected on their own assumptions and beliefs regarding planned home birth, acknowledging that these might influence the research process. To minimise this influence, a continuous exercise in reflexivity was carried out throughout the study, which included keeping a reflective diary and holding regular team discussion sessions, during which emerging interpretations were actively questioned and potential biases arising from the researchers’ position were examined. Furthermore, the team deliberately sought out data that might contradict the identified patterns, in order to reduce the risk of confirmation bias. Participants’ opinions were verified at the end of each interview to ensure accurate representation; the participants expressed their agreement, so no changes were made after verification, thus ensuring confirmability [38]. Regarding transferability, a detailed description of setting, participants, methods and data collection was provided, serving as a reference for future studies [37]. All researchers agreed with the findings.

## 3. Results

### 3.1. Participants

A total of 19 individual interviews were conducted with women who opted for a planned home birth after a previous hospital birth. Participants were aged 32–42 years. Fifteen were married and four were cohabiting. Seventeen participants were Spanish nationals and two held British–Spanish dual nationality. All participants were residing in Spain. Eleven lived in urban areas and eight in rural areas, classified according to the Spanish National Statistics Institute criteria [39]. Participants resided in four autonomous communities: Catalonia, Navarre, Balearic Islands and Andalusia. Eight had vocational training and eleven had higher education. Sixteen were in paid employment and three were unemployed. Fourteen had had two births and five had had three births. Regarding obstetric characteristics, all participants met low-risk pregnancy criteria at the time of their planned home birth. With respect to the type of previous hospital birth, nineteen had a spontaneous vaginal delivery and one had an instrumental delivery. Fifteen had experienced one planned home birth and four had experienced two planned home births (Table 1).

The qualitative analysis revealed three main themes that are summarised in Table 2.

### 3.2. Motives Related to Choosing a Planned Home Birth

This theme focuses on women’s previous experiences of childbirth in a hospital setting, considering the influence of several factors that motivated the decision to opt for a planned home birth. These factors include the treatment received from maternity healthcare professionals, the inability to make decisions during the birth process, the medicalisation of birth, and different emotions that arose during hospital birth care. The therapeutic relationship established with healthcare professionals and the lack of continuity of care were considered the precipitating factors that led the participants to opt for a planned home birth.

#### 3.2.1. Hospital Birth as a Precursor to the Decision

A common aspect mentioned by participants was the lack of involvement in decision-making during childbirth in the hospital setting which contributed to feelings of humiliation, vulnerability, and loss of bodily autonomy. In addition, most participants highlighted the multiple interventions they underwent, the restriction of movement, and the subordination to institutional rules that contributed to the loss of autonomy and dignity during the childbirth process.

*“They came into the room without introducing themselves or asking my permission. They pulled down my underwear, broke my waters, and then put up the drip. I felt completely humiliated and vulnerable … with no control over my body.” (P-2)*

*“I don’t know how many vaginal examinations they did. I was tied to the wires and the bed; they wouldn’t let me eat, and I had to urinate in a bedpan … I was under other people’s orders. For me, the care I received in hospital was very degrading.” (P-7)*

Most participants described the emotional impact they suffered during childbirth in the hospital setting due to the care they received and the complications arising from interventions that took them away from the birth they desired. Specifically, they highlighted the absence of a physiological experience, which led to feelings of sadness, emptiness, and the perception of having lost the expected memory of the birth.

*“My first birth was horrible. They induced it … I was forgotten for hours and hours, then suddenly two doctors came in and told me they would do a caesarean because I wasn’t dilating and my baby was suffering … I didn’t understand anything … It was horrible, the care was awful, I felt sad and empty, they made me feel as if they had stolen the beautiful memory of my daughter’s birth.” (P-10)*

#### 3.2.2. Lack of Continuity and Fragmented Care for Women During Childbirth

Most participants emphasised a lack of continuity of care and the fragmented care they received, characterised by the variability of healthcare professionals involved in the delivery, considering that this negatively affected the establishment of a trusting therapeutic relationship, creating an environment of mistrust and insecurity.

*“What I can’t understand is being seen by so many different midwives and gynaecologists; if the midwife doesn’t know me and I don’t know her, how am I supposed to trust her and feel safe with her?” (P-17)*

Some participants described the lack of humanisation in the process, having experienced intimidating attitudes and a lack of respect for their privacy, reinforcing the feeling of not being treated as humans.

*“At certain moments in hospital I even felt intimidated … different midwives and doctors would come into the room, pull back the sheet, and put their hand inside me without my permission. They should be clear that they are not working with furniture but with people.” (P-8)*

### 3.3. Seeking a Physiological and Humanised Birth

This theme addresses women’s need to experience a physiological and humanised birth in an intimate setting surrounded by their families, as well as the difficulty of accessing a planned home birth. It highlights women’s involvement in making free and informed decisions, their self-confidence in their ability to give birth, their satisfaction with the process, and their reliance on their own resources to be able to live this experience, which is considered rewarding and empowering for women.

#### 3.3.1. Navigating Challenges in Accessing Planned Home Birth

Some of the participants emphasised that the only information and support they received was through their own online research, midwife associations, or other women who had been through the same situation.

*“The information and support I found was through the internet, thanks to midwifery associations and other women who had given birth at home, because in the hospital no one guided me.” (P-9)*

Most women described a lack of support from healthcare professionals involved in childbirth, characterised by prejudice, stating that it was not recognised as a legitimate alternative within the healthcare system. Similarly, participants mentioned the financial effort required to have a planned home birth with a registered midwife.

*“At no point did I feel supported; on the contrary, I felt judged and criticised at every appointment. I’m sure they don’t see it as something acceptable.” (P-3)*

*“We had to cover the entire cost, and paying for a private midwife is not something everyone can afford.” (P-14)*

#### 3.3.2. Women’s Empowerment and Active Role in Childbirth

The women described the need to experience childbirth in a way that allowed them to regain control of the birth process, expressing a desire for a different experience in which they could feel involved and confident in their own abilities to give birth.

*“I wanted a different birth, I wanted to feel that I was helping my daughter come out, I wanted to trust myself; in short, I wanted a natural birth, without assistance.” (P-1)*

Some participants noted the positive impact on their self-esteem, considering home birth a transformative experience that brought empowerment and personal gratification to motherhood. In addition, they mentioned improving their self-perception as women and mothers by gaining a satisfying view of childbirth marked by early breastfeeding and the establishment of a strong bond with their baby.

*“It’s an incredible moment, hard to describe, it transformed me … I felt invincible, empowered as a woman, as a mother, and as a partner.” (P-12)*

*“As soon as she was born, we were at home, skin to skin with me, searching for the nipple. We were two in one; it was natural, beautiful. For me, it’s the best thing that can happen to a woman who has just become a mother.” (P-4)*

### 3.4. The Need to Increase Visibility and Establish Regulation for Planned Home Births

Regarding the third topic, women mentioned the lack of training for healthcare professionals on planned home births, the need to develop strategies to raise social awareness, better management of care and availability of midwives, and greater institutional support for planned home births.

#### 3.4.1. Social and Professional Awareness to Break Cultural Norms

Likewise, most women emphasised the importance of training healthcare professionals to provide clear, reliable and verified information about planned home births so that women can freely decide where to give birth.

*“There are things that could be improved. Above all, the healthcare professionals who attend you should be prepared to give you good information, otherwise how can you decide whether or not you want to have a home birth?” (P-5).*

Participants reported the need to develop dissemination strategies supported by the government and governmental organisations through various mass media such as social networks to raise social awareness and visibility regarding planned home births, as they felt that their partners, family members, or friends did not understand them because they did not follow cultural norms.

*“It’s necessary for everyone to know that it exists … they should promote it on their Facebook, Instagram … if public institutions made it visible, friends, family, and your partner would see it as normal and would understand you if you decided to opt it.” (P-18)*

#### 3.4.2. Integration of Planned Home Births into the Public Healthcare System

Most women highlighted the relevance of integrating planned home births into the PHS so that women can access this option for the birth of their babies. Some participants mentioned that incorporating this option into the PHS would facilitate accessibility and equity and eliminate inequalities among women.

*“I would improve things by having home birth included within PHSservices.” (P-14)*

*“Making it free would also mean that any woman who wants to could have a home birth, regardless of her financial means.” (P-19)*

On the other hand, some women emphasised the structural obstacles and difficulties in finding a registered midwife in certain geographical areas, sometimes having to change their place of residence during pregnancy, aspects that made them feel more vulnerable.

*“Although home birth has many advantages and that’s why I would opt it again, there are also things that make you feel vulnerable, like knowing that if I have a complication during labour and need to get to hospital quickly, nothing is organised.” (P-11)*

*“I find it heartbreaking that if you want to give birth at home you can’t, because there are no midwives in the city where you live and you have no choice but to go to another city.” (P-4)*

## 4. Discussion

The objective of this study was to explore the perceptions and experiences of Spanish women who opt for a planned home birth following a previous hospital birth, with regard to the reasons that motivated this decision and the care received during the planned home birth. After analysing the results, it was found that the main reasons why most women opted for a planned home birth after a previous hospital birth were related to the inability to make decisions about their own birth, the medicalisation of birth, and the treatment received during hospital birth by maternity healthcare professionals. In this sense, feelings of idealisation of childbirth are interrupted when women lose control during labour and the autonomy to participate in decision-making, which is associated with lower maternal satisfaction and negative birth experiences [16,40]. Similarly, coinciding with the results of the present research, previous studies highlight that the medicalisation of birth without the woman’s consent can negatively affect the childbirth experience, decreasing perceived control and confidence in her ability to give birth, perceiving childbirth as traumatic, experiencing fear or anxiety in subsequent births, leading to the search for more respectful and personalised alternative environments [5,27]. The continuity of care by the same healthcare professional is essential for building trusting relationships that improve maternal satisfaction and facilitate physiological childbirth [40,41], with the connection between midwife and woman being a key element in creating a safe environment that promotes the physiological process of birth [42]. However, the results of this study reveal fragmented care from healthcare professionals involved in maternity care, which prevents continuity of care and the establishment of a therapeutic relationship based on trust, communication and respect. These factors can act as protective factors, facilitating shared decision-making, increasing perceived safety, reducing fear and anxiety, and leading to more positive and satisfying birth experiences [16,21]. This may be due to a restrictive and paternalistic model of care that contradicts the philosophy of woman-centred care, which is essential in current obstetric practice and remains in place in most developed countries [43].

One of the most relevant aspects was the need for support and information from healthcare professionals regarding planned home births, so that women could make informed decisions about where to give birth without being stigmatised, and without having to resort to resources such as associations or the internet to find information and contact peers for support and to resolve concerns related to home births [5,21]. Previous studies indicate that most midwives support women’s right to choose home birth, while other healthcare professionals, such as general practitioners, express neutral opinions [44] and neonatologists and obstetricians have negative attitudes towards planned home births, despite supporting women’s right to choose where they give birth [1,28]. However, the results of this study reveal a lack of support from maternity healthcare professionals, including midwives, who do not respect women’s right to choose their place of birth [45]. This lack of support could be attributed to the fact that the Spanish PHS does not integrate planned home births, leading to a lack of professional knowledge and a perception of risk [5,46]. Criticism, a coercive emphasis on risk, and a lack of flexibility generate conflict and discomfort among healthcare professionals and women, encouraging them to conceal their plans and seek care outside the PHS [21], as was the case with the women in this study. In Spain, as home births are not integrated into the PHS, women must hire private midwives and bear costs that generate inequalities, strain the family economy and may limit the choice to those with resources, restricting women’s right to choose where they give birth [27,46]. In this regard, the average cost of a planned home birth in Spain ranges from 2500 to 3000 euros [47], which families must cover privately, whereas hospital births are funded by the PHS at no direct cost to women, highlighting the inequality in access to this birthing option. Paradoxically, women themselves consider that home birth could be more economical for the PHS, highlighting the contradiction between potential economic sustainability and the lack of PHS coverage for choosing this birth location [9].

The data from this study reveal that women who plan a home birth report extremely positive and empowering experiences, as well as greater satisfaction compared to hospital births, particularly highlighting professional support and participation in decision-making [5]. Planned home births allow women to experience greater self-confidence in their ability to give birth without interventions, empowerment and control over the process, allowing them to experience childbirth as a physiological event, in contrast to the hospital model where standardised protocols predominate, limiting personalisation and women’s participation [27,46]. This experience has a positive psychological impact on women as it generates favourable memories of the birth, strengthens the bond between mother and baby, and initiates breastfeeding [10,48]. In cases of previous trauma, as with the participants, it is a healing experience that restores maternal confidence [5,27].

Considering women’s right to freely choose where to give birth, the opinion and attitude of healthcare professionals involved in maternity care influence women’s choice of birth location [16]; the lack of knowledge, experience and confidence among healthcare professionals to discuss alternatives to the hospital environment makes it difficult for women to decide to opt for a planned home birth [48]. In Spain, the absence of planned home births in the PHS leads to a lack of training and experience among healthcare professionals regarding birth locations, creating a perception of risk that limits their informed support for women [46]. However, in the Netherlands, New Zealand and the United Kingdom, where planned home births are funded by the PHS, midwives are exposed to home births during their midwifery training, adopting positive attitudes that promote planned home births [43,49,50]. Therefore, training healthcare professionals on planned home births is essential to respect the preferences of families who choose this location for the birth of their baby [44]. Planned home births are considered outside of the cultural norm in Spain [5], highlighting the need to develop strategies based on the dissemination of reliable information that highlights the safety of this place of birth, helps to demystify social stereotypes and promotes understanding to encourage respect and acceptance towards women who choose planned home births [51].

In Spain, there is a lack of policies and care provision by the PHS with regard to planned home births. The PHS only offers women the option of giving birth in an obstetric unit, forcing women who opt for a planned home birth to hire private services from independently practising midwives [9]. This situation creates unequal access among women of different socioeconomic statuses, constituting an inequity in access to birth locations [23]. The care of planned home births in the current PHS needs the support of the government and government institutions to contribute to the development of protocols and resources that guarantee the safety of this place for birth [5]. In countries such as the Netherlands, the United Kingdom and Canada, where home birth is integrated into the PHS, there are explicit policies, institutional information, care coordination protocols and specific training for midwives [42]. In this regard, the inclusion of planned home births in PHS shows favourable perinatal and neonatal outcomes, comparable to those of hospital births [2]. However, studies conducted in settings without such integration report higher rates of intrapartum and neonatal adverse outcomes [52], suggesting that the safety of planned home births is closely linked to the degree of integration within the PHS. Along these lines, one study states that home birth cannot be considered safe under the current structure of the Spanish PHS, which lacks coordination and unified criteria between the different levels of care [53]. This is consistent with international evidence suggesting that variations in outcomes for planned home births are determined by the availability of established referral protocols, interprofessional coordination and the presence of midwives trained within the PHS [11]. The inclusion of planned home births in the PHS could facilitate the development and implementation of protocols, reduce the financial barriers that limit women’s access, and promote the training of healthcare professionals, potentially helping to improve the safety of this birth setting [27].

### 4.1. Limitations

This study presents several limitations which should be considered when interpreting the results and implications. Firstly, although the sample size (n = 19) is consistent with the statistical power criteria adopted in this study, it limits the scope of the findings and prevents broader generalisations. Furthermore, the study may be subject to survival bias, given that only women who successfully completed the process of planning and attending a planned home birth were included, whilst those who considered this option but ultimately withdrew or who had adverse experiences were excluded, which could have resulted in an over-representation of positive experiences. Finally, the use of a single data collection method and a single source of information represent a lack of methodological triangulation; the inclusion of multiple sources, such as midwives’ clinical records, partners’ perspectives or direct observation, could have strengthened the credibility of the findings. Moreover, the study sample consists exclusively of women from the same country, which may limit the transferability of the results to other settings with different organisational structures, particularly to countries where planned home birth is integrated into the PHS, where women’s experiences and access conditions differ substantially. Furthermore, participants came from four autonomous communities (Catalonia, Navarre, Balearic Islands and Andalusia), which limits the geographical representativeness of the results to the whole of Spain. Nevertheless, three of these communities (Catalonia, Navarre and Balearic Islands) have the highest rates of planned home birth in Spain, which facilitated access to the study population and provided contextual relevance to the findings. However, in order to obtain a representative sample of the target population, women from different geographical areas were selected for our study. The sample was relatively homogeneous (limited age range and a predominance of women with higher levels of education and/or healthcare training), which may limit the diversity of perspectives and the generalisability of the findings; future studies should include more diverse profiles. Furthermore, a potential limitation was the absence of a comparison group, as well as selection bias arising from the exclusive inclusion of women who opted for planned home births, which may limit the interpretation of the result. The inclusion of a comparison group of women who considered but ultimately did not opt for a planned home birth could have provided a more comprehensive understanding of the decision-making process. Similarly, the absence of negative or ambivalent experiences regarding planned home births could reflect a self-selection bias, whereby women with more positive experiences were more willing to participate. Another possible limitation, characteristic of the nature of qualitative research, is that data were collected through interviews, which could induce a social desirability bias, as participants may have given responses they considered socially acceptable that did not accurately reflect their experiences and perceptions. To mitigate this bias, a trained moderator was present during all interviews, and participants were offered the opportunity to review their transcripts. Furthermore, the time interval between the planned home birth and the interview (range: 2–6 months; mean: 3.8 months) may have introduced recall bias, as participants could have reinterpreted or idealised their experiences over time. Nevertheless, this interval falls within the optimal range recommended in qualitative research on birth experiences, and the inclusion criterion requiring a planned home birth within the last year limited the maximum interval to 12 months. Likewise, interviews were conducted in diverse environments, including participants’ homes, a room in the Faculty of Health Sciences, or virtual meetings. Although this flexibility supported comfort, it may have introduced variability in the interview context and influenced how participants expressed their experiences. On the other hand, the study explored the perceptions and experiences of women who had opted for a planned home birth after a previous hospital birth, and their partners were excluded. Similarly, the sample selection did not consider the experiences and perceptions of midwives who attend planned home births and hospital births. A heterogeneous sample of women, their partners, and midwives attending planned home births and hospital births would have provided different perspectives that could have enriched the results of the present study.

### 4.2. Implications for Practice

The results of this study suggest that healthcare professionals involved in maternity care could be benefit from specific training on planned home births in order to provide evidence-based and impartial information during prenatal consultations and to support informed decision-making by women. The findings also highlight the participants’ perception of the need to improve the quality of care and respectful treatment of women in order to meet their expectations and needs and increase their satisfaction with the birth experience. Based on the information provided by the women, midwives who attend planned home births could consider developing clear and standardised care protocols to ensure the safety of the mother and baby. Furthermore, the experiences reported by participants suggest that exploring the feasibility of integrating planned home births into the PHS could contribute to improving access and equity for women who choose this birth location in Spain, although further research with larger and more diverse samples is needed to inform such policy decisions. Similarly, the findings suggest that healthcare institutions could consider promoting shared decision-making between women and healthcare professionals involved in maternity care regarding birth location, respecting women’s autonomy and informed preferences.

### 4.3. Further Research

Future studies could develop qualitative research that includes the perspective and experience of other healthcare professionals involved in the hospital birth process, as well as midwives who attend planned home births and hospital births, in order to obtain a deeper understanding of this phenomenon. Similarly, exploring the experiences and perceptions of the partners of women who opt for a planned home birth would allow for a deeper understanding and identification of the underlying reasons behind the shared decision to choose a planned home birth. Furthermore, studies are needed that explore maternal and neonatal outcomes in planned home births to ensure their safety and to promote the humanisation of childbirth and its integration into the PHS. Likewise, future studies should therefore include recruitment strategies that facilitate the participation of women with a range of experiences.

## 5. Conclusions

Women who opted for a planned home birth after a previous hospital experience identified the medicalisation of birth, lack of participation in decision-making and the treatment they received as the main reasons for choosing this location for the birth of their baby. Women perceived planned home birth as a transformative, satisfying, and empowering experience that allowed them to regain control of the process, strengthen the bond with their baby, and initiate breastfeeding. According to the participants’ experiences, the lack of support from health professionals, the absence of regulation and the economic cost of private services were perceived as barriers that create inequalities in access to planned home births, limiting women’s right to choose where they give birth. Participants expressed the need to train health professionals on home birth, develop outreach strategies to raise awareness of this birth location as safe, and establish protocols to ensure safety and coordination with the PHS. From the perspective of the women interviewed, the integration of planned home birth into the PHS could contribute to ensuring equity in access to this birth option for all women, allowing them to make an informed choice about where they want to give birth to their baby.

## Figures and Tables

**Table 1:** Sociodemographic characteristics of participants.

Participant	Age	Marital Status	Nationality	Place of Residence	Education	Employment	Region	Obstetric Risk	Type of Previous Hospital Birth	Number of Births	Home Birth	Hospital Birth
P-1	34	Married	SP	Urban	VT	Shop assistant	Catalonia	Low	SVD	2	1	1
P-2	42	Married	SP	Urban	VT	Auxiliary nurse	Andalusia	Low	SVD	2	1	1
P-3	32	Married	SP	Urban	HE	Lawyer	Catalonia	Low	SVD	2	1	1
P-4	38	Married	SP	Rural	VT	Osteopath	Catalonia	Low	SVD	3	2	1
P-5	40	Married	B-SP	Rural	HE	Nutritionist	Balearic Islands	Low	SVD	3	2	1
P-6	32	Cohabiting	SP	Rural	VT	Unemployed	Catalonia	Low	SVD	2	1	1
P-7	33	Married	SP	Rural	VT	Pilates instructor	Balearic Islands	Low	SVD	2	1	1
P-8	35	Married	SP	Urban	HE	Teacher	Catalonia	Low	SVD	2	1	1
P-9	38	Married	SP	Urban	HE	Nurse	Andalusia	Low	SVD	2	1	1
P-10	39	Cohabiting	SP	Urban	HE	Teacher	Navarre	Low	SVD	3	2	1
P-11	34	Married	SP	Urban	HE	Administrative	Navarre	Low	SVD	2	1	1
P-12	33	Married	SP	Rural	VT	Unemployed	Andalusia	Low	SVD	2	1	1
P-13	33	Cohabiting	B-SP	Rural	HE	Teacher	Balearic Islands	Low	SVD	2	1	1
P-14	41	Married	SP	Urban	VT	Unemployed	Catalonia	Low	SVD	3	2	1
P-15	33	Cohabiting	SP	Urban	HE	Teacher	Catalonia	Low	SVD	2	1	1
P-16	39	Married	SP	Rural	VT	Teacher	Andalusia	Low	SVD	3	1	2
P-17	36	Married	SP	Rural	HE	Sexologist	Navarre	Low	SVD	2	1	1
P-18	35	Married	SP	Urban	HE	Social worker	Catalonia	Low	SVD	2	1	1
P-19	41	Married	SP	Urban	HE	Midwife	Catalonia	Low	SVD	2	1	1

Note. P, participant; SP, Spanish; B-SP, British–Spanish dual nationality; VT, vocational training; HE, higher education; SVD, spontaneous vaginal delivery.

**Table 2:** Themes, subthemes, codes and representative quotes.

Themes	Subthemes	Codes	Representative Quotes
Motives related to choosing a planned home birth	Hospital birth as a precursor to the decision	Loss of autonomy and decision-making during hospital birth, unnecessary medical interventions and restriction of movement, feelings of humiliation, vulnerability and loss of dignity Emotional impact: sadness, emptiness and sense of stolen birth experience Absence of a physiological birth experience	‘I couldn’t take it anymore and had to ask for an epidural. I think that if they hadn’t done so many things to me, I could have endured it and had a natural birth like I wanted.’ (P-19).
	Lack of continuity and fragmented care for women during childbirth	Multiple healthcare professionals during labour, inability to establish a trusting therapeutic relationship, environment of mistrust and insecurity, lack of humanisation and intimidating attitudes, disregard for privacy and bodily autonomy	‘For me, the first thing that needs to be improved is that the same midwife who has been looking after you throughout your pregnancy and has known you since you were just a few weeks pregnant should be the one who helps you give birth… it’s logical.’ (P-13).
Seeking a physiological and humanised birth	Navigating challenges to accessing planned home birth	Lack of professional support and guidance from healthcare professionals, prejudice and stigmatisation from maternity care providers, self-directed information seeking through internet and associations, peer support from other women with home birth experience, financial burden of private midwifery services	‘I didn’t feel supported by either the midwife or the doctors… I felt like a freak and a bad mother for not choosing what was best for my baby. They made me feel selfish.’ (P-16).
	Women’s empowerment and active role in childbirth	Desire to regain control over the birth process, self-confidence in the ability to give birth, transformative and empowering experience, positive impact on self-esteem and self-perception, early breastfeeding and mother–baby bonding	‘Above all, it helped me to believe in myself more, to feel like an independent and capable woman.’ (P-6).
The need to increase visibility and establish regulation for planned home births	Social and professional awareness to break cultural norms	Need for professional training on planned home birth, evidence-based information for informed decision-making, dissemination strategies through mass media and social networks, institutional support for visibility, lack of understanding from partners, family and social environment	‘I believe that planned home births need to be made more visible, they need to be publicised, and there is no better way to do this than through healthcare institutions.’ (P-15).
	Integration of planned home births into the Public Healthcare System	Need for inclusion of home birth in Public Health System, accessibility and equity for women, elimination of economic inequalities, structural obstacles and geographical scarcity of midwives, lack of coordination and transfer protocols with hospitals	‘During my home birth, I ended up going to the hospital because I was not dilating… when I arrived, I heard arguments… I felt that there was little organisation and that they were not coordinated.’ (P-18).

## Data Availability

Data are available upon request from the corresponding author. The data are not publicly available due to privacy and ethical restrictions.

## Supplementary Materials

The following supporting information can be downloaded at https://www.mdpi.com/article/10.3390/nursrep16040147/s1, Standards for Reporting Qualitative Research (SRQR); Table S1: Interview protocol.

## Public Involvement Statement

There was no public involvement in any aspect of this research.

## Guidelines and Standards Statement

This manuscript was drafted against the Standards for Reporting Qualitative Research (SRQR), used to report this study [33].
