# Supplementary material for: Experiences of Women Who Opt for a Planned Home Birth After a Previous Hospital Birth: A Qualitative Study
Source: Nurs Rep. 2026 Apr 21;16(4):147. doi: 10.3390/nursrep16040147 (PMC13118726; doi:10.3390/nursrep16040147)
Supplement: Supplementary file 1 [file nursrep-16-00147-s001.zip › nursrep-4224185-supplementary.pdf]

Supplementary Table S1. Interview protocol

Phase	Topics	Content/example questions
Introduction	Aim of study	We are conducting a study to understand the perceptions and experiences of women who choose a planned home birth after having previously given birth in a hospital. We believe that their experience and perception is a vital insight to explore new perspectives on planned home births, to make them more visible and to improve the quality of care of this care process.
	Purpose	Carry out and publish research based on perceptions and experiences of Spanish women who choose to have a planned home birth after a previous hospital birth, regarding the reasons behind this decision and the care received during the planned home birth
	Ethical issues	Inform about volunteering, recording, consent, possibility of withdrawing.
Opening	Introductory question	Please, tell me your perceptions and experiences to opt for a planned home birth after a previous hospital birth.
Development	Conversation guide	Have you done planned home births, could you describe your experience, or tell us what you know about planned home births?
		How would you describe your experience during your previous hospital birth? What aspects did you value positively and which ones negatively?
		In what way do you think the planned home birth influenced your physical and emotional wellbeing compared to your previous hospital experience?
		What were the main reasons that led you to opt for a planned home birth after having had a hospital birth?
		What difficulties did you encounter as a woman in order to carry out a planned home birth in Spain?
		How would you rate the care and support provided by the midwife during your planned home birth? What aspects of her intervention would you highlight?
		What changes or improvements do you think would be necessary for planned home birth to be included and regulated within the public health system in Spain?

Closing	Last question	Would you like to add anything else that you consider relevant? Anything to clarify before we end?
	Appreciation	Remind them of how their information will be used and tell them they can contact us if you have any queries<br>Once the research is completed, we will send you a copy
	Acknowledgements	Thank you for your time and interest. Certainly, your statement will be useful for the research
Translation	Retro-translations	The interview statements will be translated into English by bilingual researchers. They will then be back-translated into Spanish by bilingual researchers and compared with the original transcripts to ensure accuracy.

Standards for Reporting Qualitative Research (SRQR)*

http://www.equator-network.org/reporting-guidelines/srqr/

Title and abstract

	Page/line no(s).
**Title** - Concise description of the nature and topic of the study Identifying the study as qualitative or indicating the approach (e.g., ethnography, grounded theory) or data collection methods (e.g., interview, focus group) is recommended	1
**Abstract** - Summary of key elements of the study using the abstract format of the intended publication; typically includes background, purpose, methods, results, and conclusions	1

Introduction

**Problem formulation** - Description and significance of the problem/phenomenon studied; review of relevant theory and empirical work; problem statement	2-4
**Purpose or research question** - Purpose of the study and specific objectives or questions	5

Methods

**Qualitative approach and research paradigm** - Qualitative approach (e.g., ethnography, grounded theory, case study, phenomenology, narrative research) and guiding theory if appropriate; identifying the research paradigm (e.g., postpositivist, constructivist/ interpretivist) is also recommended; rationale**	5
**Researcher characteristics and reflexivity** - Researchers' characteristics that may influence the research, including personal attributes, qualifications/experience, relationship with participants, assumptions, and/or presuppositions; potential or actual interaction between researchers' characteristics and the research questions, approach, methods, results, and/or transferability	6
**Context** - Setting/site and salient contextual factors; rationale**	
**Sampling strategy** - How and why research participants, documents, or events were selected; criteria for deciding when no further sampling was necessary (e.g., sampling saturation); rationale**	6
**Ethical issues pertaining to human subjects** - Documentation of approval by an appropriate ethics review board and participant consent, or explanation for lack thereof; other confidentiality and data security issues	8
**Data collection methods** - Types of data collected; details of data collection procedures including (as appropriate) start and stop dates of data collection and analysis, iterative process, triangulation of sources/methods, and modification of procedures in response to evolving study findings; rationale**	6-7

**Data collection instruments and technologies** - Description of instruments (e.g., interview guides, questionnaires) and devices (e.g., audio recorders) used for data collection; if/how the instrument(s) changed over the course of the study	
**Units of study** - Number and relevant characteristics of participants, documents, or events included in the study; level of participation (could be reported in results)	6
**Data processing** - Methods for processing data prior to and during analysis, including transcription, data entry, data management and security, verification of data integrity, data coding, and anonymization/de-identification of excerpts	7-8
**Data analysis** - Process by which inferences, themes, etc., were identified and developed, including the researchers involved in data analysis; usually references a specific paradigm or approach; rationale**	7-8
**Techniques to enhance trustworthiness** - Techniques to enhance trustworthiness and credibility of data analysis (e.g., member checking, audit trail, triangulation); rationale**	8-9

Results/findings

**Synthesis and interpretation** - Main findings (e.g., interpretations, inferences, and themes); might include development of a theory or model, or integration with prior research or theory	9-16
**Links to empirical data** - Evidence (e.g., quotes, field notes, text excerpts, photographs) to substantiate analytic findings	9-16

Discussion

**Integration with prior work, implications, transferability, and contribution(s) to the field -** Short summary of main findings; explanation of how findings and conclusions connect to, support, elaborate on, or challenge conclusions of earlier scholarship; discussion of scope of application/generalizability; identification of unique contribution(s) to scholarship in a discipline or field	17-20
**Limitations** - Trustworthiness and limitations of findings	20

Other

**Conflicts of interest** - Potential sources of influence or perceived influence on study conduct and conclusions; how these were managed	21
**Funding** - Sources of funding and other support; role of funders in data collection, interpretation, and reporting	21

*The authors created the SRQR by searching the literature to identify guidelines, reporting standards, and critical appraisal criteria for qualitative research; reviewing the reference lists of retrieved sources; and contacting experts to gain feedback. The SRQR aims to improve the transparency of all aspects of qualitative research by providing clear standards for reporting qualitative research.

**The rationale should briefly discuss the justification for choosing that theory, approach, method, or technique rather than other options available, the assumptions and limitations implicit in those choices, and how those choices influence study conclusions and transferability. As appropriate, the rationale for several items might be discussed together.

Reference:

O'Brien BC, Harris IB, Beckman TJ, Reed DA, Cook DA. **Standards for reporting qualitative research: a synthesis of recommendations.** *Academic Medicine*, Vol. 89, No. 9 / Sept 2014 DOI: 10.1097/ACM.0000000000000388

UNIVERSIDAD DE ALMERÍA

6. **<u>Consentimiento informado</u>**

CONSENTIMIENTO INFORMADO POR ESCRITO DEL PACIENTE

Yo (Nombre y Apellidos) ....

• He leído el documento informativo que acompaña a este consentimiento (Información al Paciente)

• He podido hacer preguntas sobre el estudio.

• He recibido suficiente información sobre el estudio. He hablado con el profesional investigador: [ANONIMIZED]

• Comprendo que mi participación es voluntaria y soy libre de participar o no en el estudio.

• Se me ha informado que todos los datos obtenidos en este estudio serán confidenciales y se tratarán conforme establece la Ley Orgánica de Protección de Datos de Carácter Personal 15/99.

• Se me ha informado de que la información obtenida sólo se utilizará para los fines específicos del estudio.

Comprendo que puedo retirarme del estudio:

• Cuando quiera
• Sin tener que dar explicaciones

Presto libremente mi conformidad para participar en el proyecto titulado "*Provider Attitudes towards Planned Home Birth (PAPHB) Scale: Adaptation and validation*."

Firma del participante (o representante legal en su caso)

Nombre y apellidos:……………………………………..

Firma del profesional investigador

Nombre y apellidos: [ANONIMIZED]
Enfermera Obstétrico-Ginecológica (Matrona) e investigadora principal.

Fecha: ……………………………….   Fecha: ……………………….
